# A ‘Terror of Tyrannosaurs’: The First Trackways of Tyrannosaurids and Evidence of Gregariousness and Pathology in Tyrannosauridae

**DOI:** 10.1371/journal.pone.0103613

**Published:** 2014-07-23

**Authors:** Richard T. McCrea, Lisa G. Buckley, James O. Farlow, Martin G. Lockley, Philip J. Currie, Neffra A. Matthews, S. George Pemberton

**Affiliations:** 1 Peace Region Palaeontology Research Centre, Tumbler Ridge, British Columbia, Canada; 2 Department of Earth and Atmospheric Sciences, University of Alberta, Edmonton, Alberta, Canada; 3 Department of Biological Sciences, University of Alberta, Edmonton, Alberta, Canada; 4 Department of Geosciences, Indiana-Purdue University, Fort Wayne, Indiana, United States of America; 5 Dinosaur Trackers Research Group, University of Colorado at Denver, Denver, Colorado, United States of America; 6 Bureau of Land Management, Denver, Colorado, United States of America; University of Pennsylvania, United States of America

## Abstract

The skeletal record of tyrannosaurids is well-documented, whereas their footprint record is surprisingly sparse. There are only a few isolated footprints attributed to tyrannosaurids and, hitherto, no reported trackways. We report the world’s first trackways attributable to tyrannosaurids, and describe a new ichnotaxon attributable to tyrannosaurids. These trackways are from the Upper Cretaceous (Campanian - Maastrichtian) of northeastern British Columbia, Canada. One trackway consists of three tridactyl footprints, and two adjacent trackways consist of two footprints each. All three trackways show animals bearing southeast within an 8.5 meter-wide corridor. Similarities in depth and preservation of the tyrannosaurid tracks indicate that these three trackways were made by track-makers walking concurrently in the same direction. These trackways add significantly to previous osteology-based hypotheses of locomotion and behavior in Tyrannosauridae by providing ichnologic support for gregariousness in tyrannosaurids, and the first record of the walking gait of tyrannosaurids.

## Introduction

**Figure 1:**
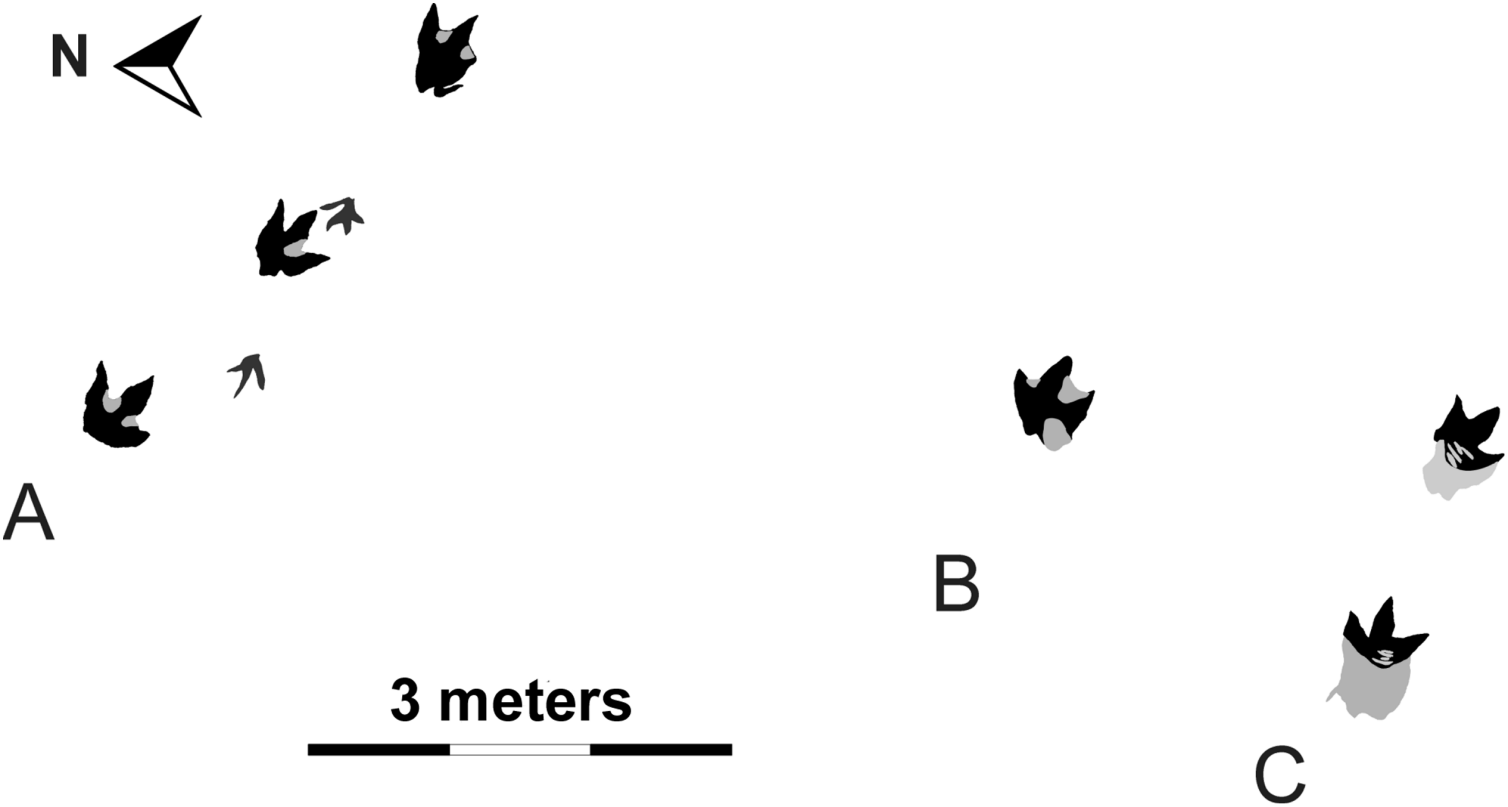
Trackway map. A portion of the tracksite showing three parallel trackways of *Bellatoripes fredlundi* and a partial trackway of a smaller theropod *Saurexallopus cordata*. Trackway A: PRPRC 2011.11.001; Trackway B: PRPRC 2012.04.002; Trackway C: PRPRC 2012.04.003. This trackway map was produced by tracings taken from the site, but outlines of the prints were produced by studying the silicone moulds of all the trackways at the Peace Region Palaeontology Research Centre (PRPRC). Trackway B has two tracks (only the first one is figured, the second is a pace length in front), but a debris slide covered the second print shortly after discovery at the end of the field season.

Reports of footprints attributable to tyrannosaurids are rare [1] and although footprints of tyrannosaurids occur in Mongolia [2], the western United States [1], [3], [4] and western Canada [5], [6], [7], [8], they were only known from single footprint occurrences. The lack of trackways attributable to tyrannosaurids left a conspicuous gap in locomotor and behavioral data for this group. Hypotheses on locomotion [9], [10], [11], [12], [13] and behavior [14], [15] of tyrannosaurids have been based solely on osteological material. There has never been an opportunity to test these hypotheses against a preserved record of tyrannosaurid movement, including possible group behavior. Here, we describe the first trackways (as opposed to rare and controversial isolated footprints) attributable to tyrannosaurids, as a new ichnotaxon (ichnogenus and ichnospecies) within a newly established ichnofamily. These trackways occur in the Upper Cretaceous (upper Campanian - lower Maastrichtian) Wapiti Formation in northeastern British Columbia. Three trackways (Fig. 1) occur on a 60 m×3 m bedding plane exposure along with individual prints of large ornithopods (*Hadrosauropodus* isp.) and smaller theropods (*Saurexallopus cordata*) [16]. The track-layer is composed of fine-grained silty-sandstone with significant clay content and visible carbonized plant fragments. It is likely that the high clay content of the substrate allowed sediment displaced by the large theropod feet to be compacted rather than extruded to form sediment rims around the footprint, which can obscure footprint features. The compaction of the clay was probably an important factor in preventing deeply impressed tyrannosaurid prints from collapsing after the removal of the track-makers’ feet.

Despite the large size of the footprints of the track-makers, there were only minor disturbances of sediment between digit impressions, and no detectable development of displacement rims around the tracks. The track-bearing substrate was the original surface as evidenced by the presence of skin impressions (consisting of small, indistinctly-shaped tubercles) and striations in several of the tyrannosaurid tracks. Skin impressions also occur in some of the *Hadrosauropodus* isp. tracks on the same surface several meters away. The track-bearing bed was observed to be overlain by a 30 cm thick layer of kaolinite. At present it is unclear if this kaolinite layer is uniformly distributed over the entire track-bearing surface.

**Figure 2:**
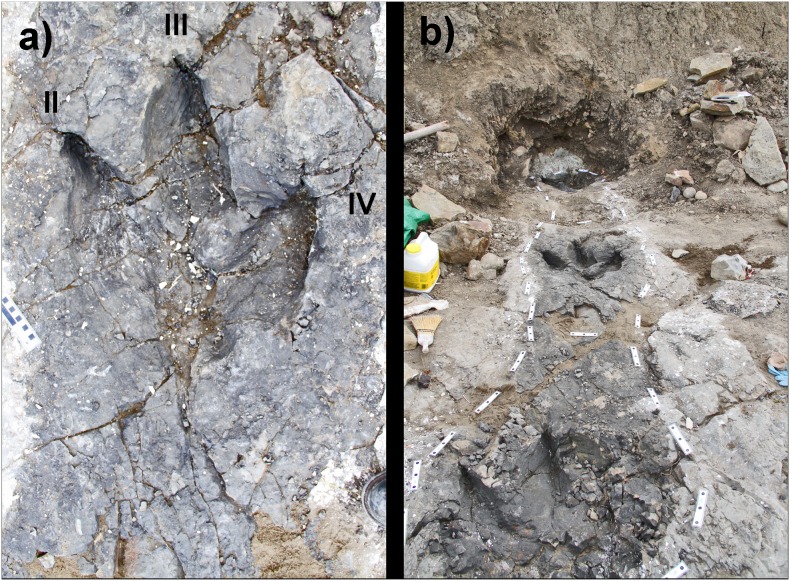
*In situ* Trackway A images. **a)** Print #2 of Trackway A (*in situ*) - PRPRC 2011.11.001 (right); **b)** Trackway A (*in situ*) view to the east of prints #1–3. Note the thick layer of kaolinite in the freshly excavated area in front of print #3.

**Figure 3:**
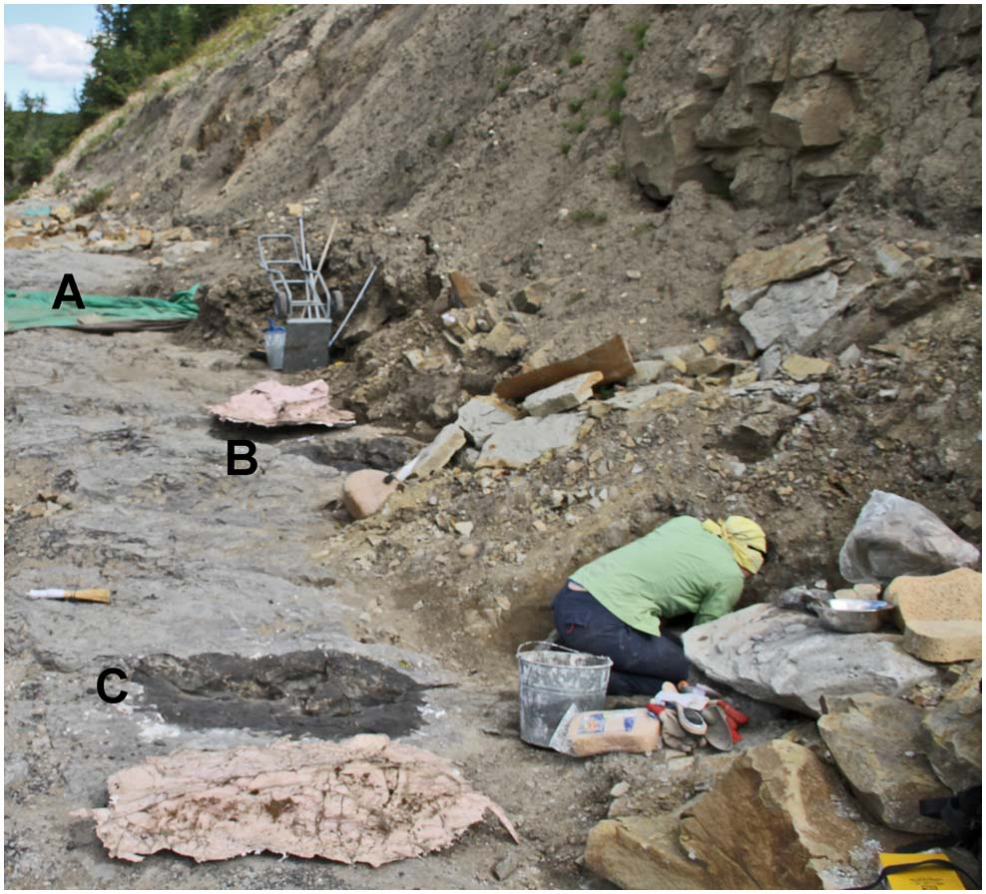
Excavation of Tracksites (A: Trackway A; B: Trackway B; C: Trackway C). View toward the north from near Trackway C (foreground with LGB excavating and silicone mould of print #1 at the bottom of the figure), Trackway B is located in the center of the image and the silicone mould of Trackway B, print #1 is visible. Trackway A is covered in a green tarp near the top left of the image. The headings of the trackways are from left to right with other tracks likely buried by sediments forming a steep cliff.

The first two prints of Trackway A were discovered in early October, 2011 by Mr. Aaron Fredlund, a local guide-outfitter. The third print was excavated by Peace Region Palaeontology Research Centre (PRPRC) staff and volunteers in late October of the same year (Fig. 2). Trackway A consists of three large tridactyl footprints with several characteristics that allow identification of the track-maker as a large theropod. These include large terminal claw impressions, FL (footprint length) > FW (footprint width), the three digit impressions extending roughly the same distance distally from the footprint heel (but with III longest and IV generally longer than II), presence of a clawed hallux impression on one of the prints, as well as having long pace and stride with a high pace angulation. Two additional trackways (Trackway B and C) were discovered by excavating in the area immediately south of Trackway A in August, 2012 (Fig. 3).

## Materials and Methods

### Molding and Measurements

**Figure 4:**
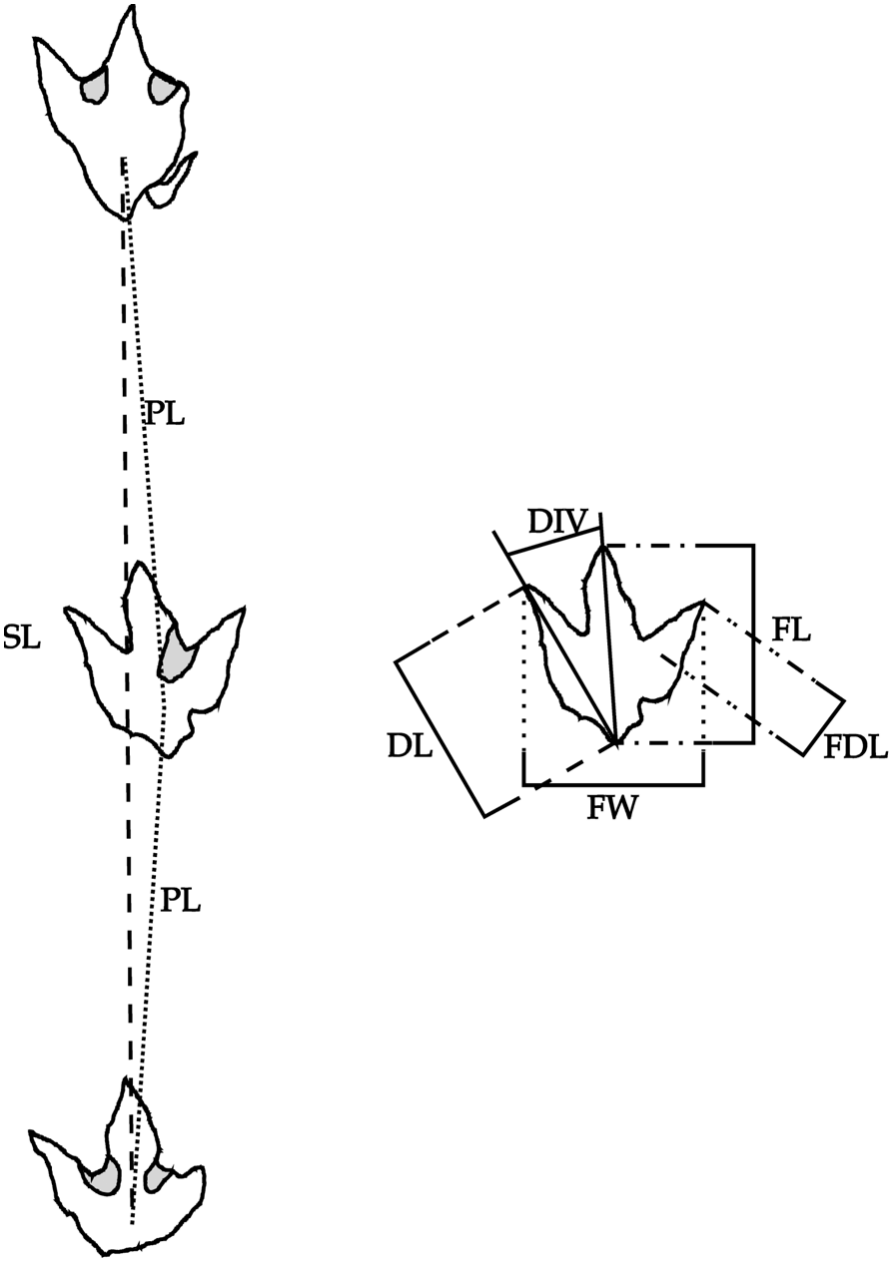
Methods of trackway and footprint measurements used in this study. DIV , digit divarication; **DL**, digit length; **FDL**, free digit length; **FL**, footprint length; **FW**, footprint width; **PL**, pace length; **SL**, stride length.

No permits were required for this study. The original ‘Trackway A’ (PRPRC 2011.11.001) was molded (PRPRC 2011.11.001M) using platinum-cure silicone reinforced with a sectional plaster support jacket constructed at the site. A 1∶1 replica of the trackway was cast (PRPRC 2011.11.001MC) using fiberglass-reinforced plaster (FGR 95). Linear and angle data were compiled from measurements taken directly from the silicone mold (Fig. 4). Measurements were taken of the original *in situ* trackway, but due to the degree to which the digit impressions undercut the track-bearing surface, these measurements were not accurate. The most accurate measurements were made possible through the study of the replica mold (PRPRC 2011.11.001M), where all track morphologies and trackway features are accessible to workers. Measurements of Trackways B and C were made from their silicone molds (PRPRC 2012.04.002M and PRPRC 2012.04.003M, respectively).

Images for photogrammetric analysis were obtained with a Canon EOS 7D. Resulting image files were processed in Agisoft Photoscan Professional (v 1.0.4) and Cloud Compare (v 2.5.3).

A new quantitative method for comparing the robustness of tracks was developed by manually measuring the area (cm^2^) of individual footprints (excluding the hallux impression and extra-morphological features, e.g. caudal-lateral drag-marks, etc.), and calculating a ratio (Footprint Area to Length Ratio; units in cm) by dividing the footprint area (cm^2^) by its length (cm). Creating an area to length ratio allows for quantitative comparisons of the overall robustness of tracks of similar morphology (i.e. tridactyl tracks) of all sizes. The Footprint Area to Length Ratio uses Thulborn’s [17] track surface area calculation while accounting for the track surface area occupied per linear track length, which can be compared across dinosaurian ichnotaxa.

### Calculations of Track-Maker Velocity and Age

Relative velocities of the track-makers were calculated using formulae from Thulborn and Wade [18] and Alexander [19] using the formula
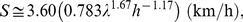
(1)with λ as stride length in centimeters, and *h* as height at the hip, or pelvic limb length, in centimeters.

Thulborn and Wade [18] refined the methodology used to calculate height at the hip (*h*) of bipedal dinosaurs for use in calculating relative velocity after Alexander [19], who suggests that hip height (*h)* ∼− 4.0X_footprint length(FL)_; however, see Alexander [19] and Coombs [20] for variations of this ratio. Thulborn and Wade [18] account for allometry in large-sized theropods by using linear regression analyses of osteometric data to solve for *h* using footprint length (FL) for large theropods (their “carnosaurs”):
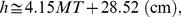
(2)with metatarsus proximodistal length (MT) considered a proxy for footprint length (FL) in large theropods.

There are sufficient osteometric data available to tailor this hip height equation specifically for tyrannosaurids. This provides the opportunity to customize the existing equations to determine the relative size and velocity of the late Campanian - early Maastrichtian tyrannosaurid track-makers, including the track-makers of *Bellatoripes fredlundi*, as well as estimate the approximate age [21] of the track-makers by determining the relationship between their femur lengths and footprint lengths.

**Figure 5:**
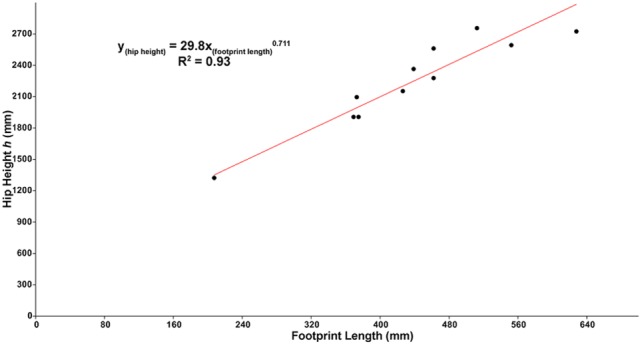
Footprint length (digit III) versus hip height (*h*, leg length) of *Albertosaurus*, *Gorgosaurus*, and *Daspletosaurus*. Graphical results and best-fit regression line for footprint length (FL, calculated at the proximodistal lengths of digit III phalanges) compared to leg length as calculated from the sum of the proximodistal femur, tibia and astragalus, and metatarsal III lengths of late Campanian - early Maastrichtian tyrannosaurids *Albertosaurus*, *Gorgosaurus*, *Daspletosaurus* [21], [25]. All data are in millimeters (mm), and are unadjusted [18]. Five percent was added to all totaled lengths to account for anatomical unknowns. Standard error for footprint length +/–33.3 mm; hip height +/–129 mm.

**Figure 6:**
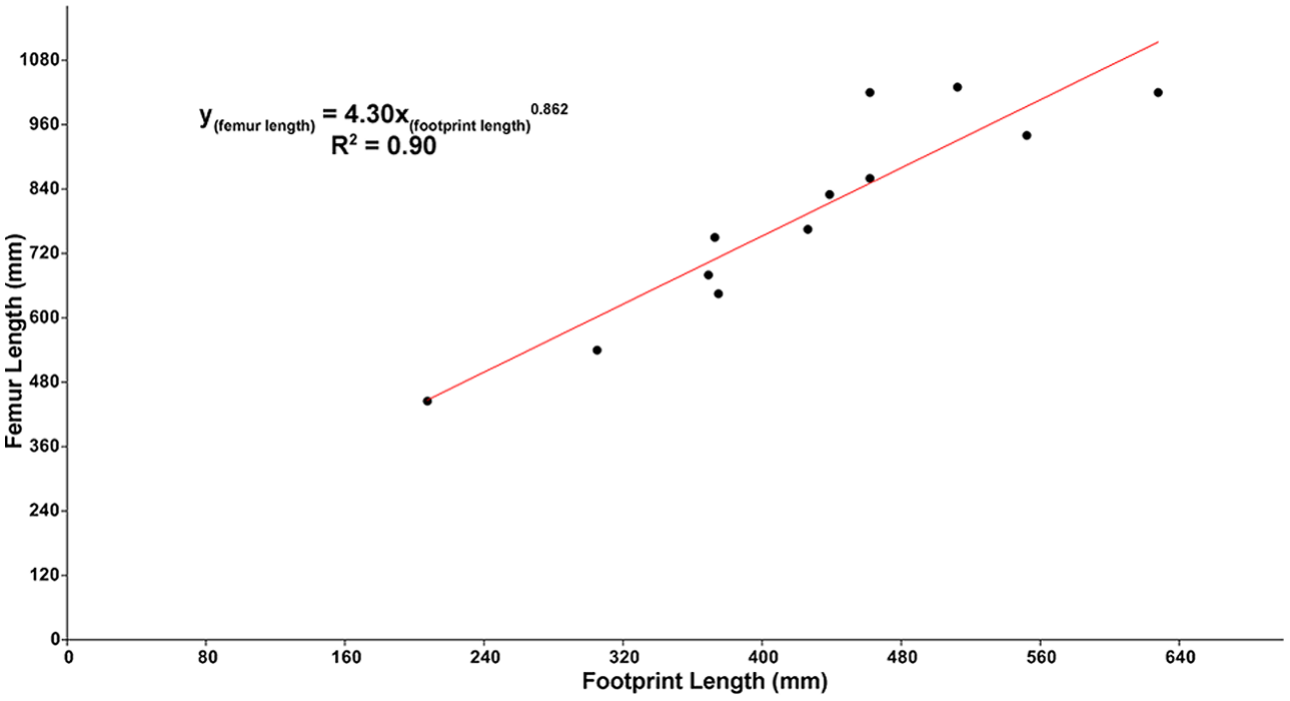
Digit III length versus femur length for *Albertosaurus*, *Gorgosaurus*, and *Daspletosaurus*. Graphical results and best-fit line for comparing footprint length FL (digit III length, as calculated by totaling the proximodistal lengths of digit III phalanges and adding 5% to the total length to account for anatomical unknowns) to osteologic femur length (*y*) for late Campanian - early Maastrichtian tyrannosaurids *Albertosaurus*, *Gorgosaurus*, *Daspletosaurus* [22]. The calculated *y* can then be used to estimate age of the track-maker using the methods of Erickson *et al.* [21], [25]. All data are in millimeters (mm), and are unadjusted [18]. Standard error for footprint length +/–32.3 mm; femur length +/–55.7 mm.

Following the methodology of Thulborn and Wade [18], and Currie [22], hindlimb data [22] from specimens of *Albertosaurus sarcophagus*, *Gorgosaurus libratus*, and *Daspletosaurus torosus* were graphed to find the best fit line for the data. Data were not log_10_ transformed [18]. Least-squares regression was used to find the best-fit lines for the bivariate comparisons of data. The power equation (y = ax^b^) produced the line that best fit the data with the highest coefficient of determination (R^2^) value (Figs. 5–6) for all graphs.

Thulborn and Wade [18] address the addition of anatomical unknowns (e.g. cartilage, tendons, keratinous sheaths, and other soft tissues such as distal metatarsal pad impressions) to limb length, but do not account for them in their calculations on the basis that these unknowns would not greatly alter the general conclusions. However, since footprints and trackways are a result of the combination of osteology and these anatomical unknowns, these unknowns need to be considered before using an ichnologic specimen to derive *h*, relative velocity, or estimated age. Thulborn [23] cites Lambe’s [24] observations on a specimen of *Gorgosaurus libratus* to determine that adding 5% to the length obtained from measuring the bones of the hind limb reasonably accounts for the anatomical unknowns in large theropods. The regression was calculated to determine the quantitative relationship between footprint length and height at the hip (leg length) of *Albertosaurus*, *Gorgosaurus*, and *Daspletosaurus*. First, we consider the osteologic FL of digit III (which includes the straight line end-to-end measurements of phalanges III-1, III-2, III-3, and III-4 plus 5% of the total) as a proxy for ichnologic FL as measured from the footprint (Fig. 5). Digit III length is calculated by summing the lengths of the pedal phalanges and adding 5% to the total:
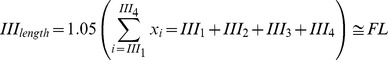
(3)


Next, the regression was made between digit III length and leg length, calculated by adding the proximodistal lengths of the femur (F), tibiotarsus (T), and metatarsus (MT). Tibia and astragalus length was measured as the proximodistal length of the tibia and the astragalus combined. The proximodistal length of the metatarsus [22], which was measured from the proximal end of the metatarsus to the distal end of metatarsal III, was used for MT when calculating leg length. Anatomical unknowns were accounted for by adding 5% to the total measured lengths of the hind limbs.
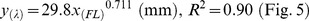
(4)


Tests of this equation using known lengths of hind limb bones of tyrannosaurids [22] yielded results between 95%–111% of the known lengths.

To determine the estimated age of the track-makers, the quantitative relationship between FL and osteologic femur length was determined using linear regression between the proximodistal length digit III plus 5% and femur lengths:
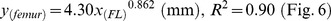
(5)


Femur length (y) can then be entered into Erickson *et al.*’s [21] equation to calculate age:
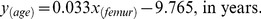
(6)


At this time we are satisfied with Erickson *et al.*’s [21] calculations, as the accuracy of the alterations made by Myhrvold [25] has yet to be assessed.

### Nomenclatural Acts

The electronic edition of this article conforms to the requirements of the amended International Code of Zoological Nomenclature, and hence the new names contained herein are available under that Code from the electronic edition of this article. This published work and the nomenclatural acts it contains have been registered in ZooBank, the online registration system for the ICZN. The ZooBank LSIDs (Life Science Identifiers) can be resolved and the associated information viewed through any standard web browser by appending the LSID to the prefix “http://zoobank.org/”. The LSID for this publication is: urn:lsid:zoobank.org:pub:0D77CC19-3DC9-4F8D-ADA0-59B3F9D9580C. The electronic edition of this work was published in a journal with an ISSN, and has been archived and is available from the following digital repositories: PubMed Central and LOCKSS.

**Systematic Ichnology**
Dinosauromorpha Benton, 1984
Dinosauriformes Novas, 1992
Dinosauria Owen, 1841
Theropoda Marsh, 1881;
Coelurosauria Gauthier, 1986;
Tyrannosauroidea Walker, 1964;
Tyrannosauridae Osborn, 1905
Ichnofamily Tyrannosauripodidae ichnofam. nov.
(urn:lsid:zoobank.org:act:C2F3EC7D-71A5-4A50-9AE7-62500EDB2000).

### Diagnosis

Large functionally tridactyl, mesaxonic tracks with distal metatarsal pad impression; may have craniomedially-directed hallux impression; footprint length greater than width; robust footprint with thick digits; generally lacking distinct digital pad impressions. Digit impressions thickened proximally, strongly tapering distally and terminating in acuminate claw impressions. Trackway narrow with slight inward rotation of pes towards midline; pace length close to 175 cm or greater; stride length close to 350 cm or greater.

### Type ichnogenus

*Tyrannosauripus* Lockley and Hunt, 1994 [4].

### Referred specimens

*Tyrannosauripus pillmorei* Lockley and Hunt [4] (CU-MWC225.1), unnamed print from Nemegt Formation MPD 100F/12 [2], print TMP 81.34.1 from the Belly River Group in Dinosaur Provincial Park, Alberta [6] and large theropod tracks from the Campanian - Maastrichtian Wapiti Formation of British Columbia (PRPRC 2004.08.001, PRPRC 2011.11.001, PRPRC 2011.11.001M, PRPRC 2011.11.001MC, PRPRC 2012.04.002, PRPRC 2012.04.002M, PRPRC 2012.04.003, PRPRC 2012.04.003M) described by McCrea *et al.*, herein.

**Figure 7:**
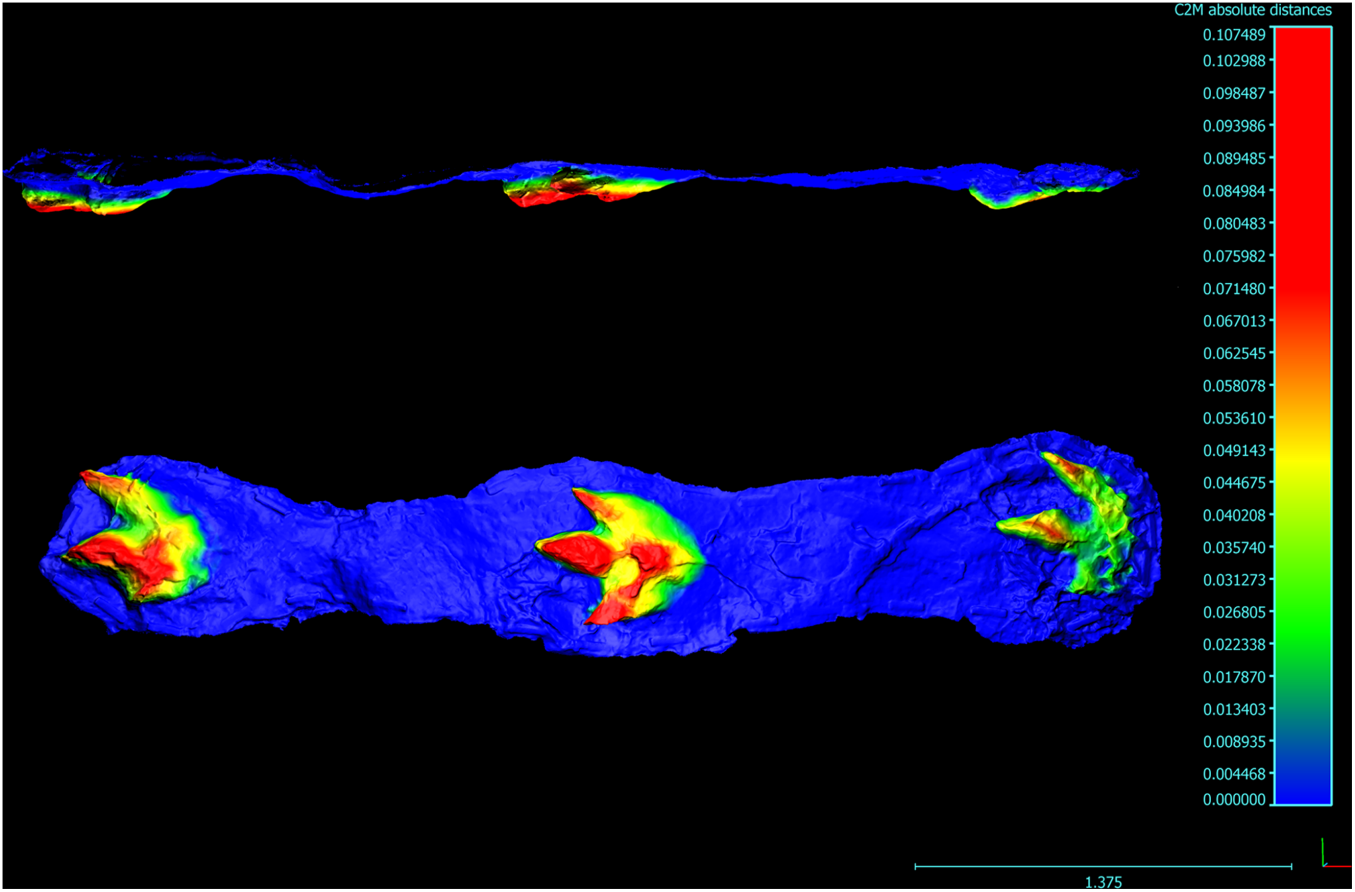
Photogrammetic image of *Bellatoripes fredlundi* Holotype (Trackway A). Figure rendered from images of the silicone mould (PRPRC 2011.01.001M). Lateral view (top) and plan view (bottom). Note that the topographic profile for the lateral view is reversed in this orientation. Topographic profile scale and linear scale are in meters.

**Figure 8:**
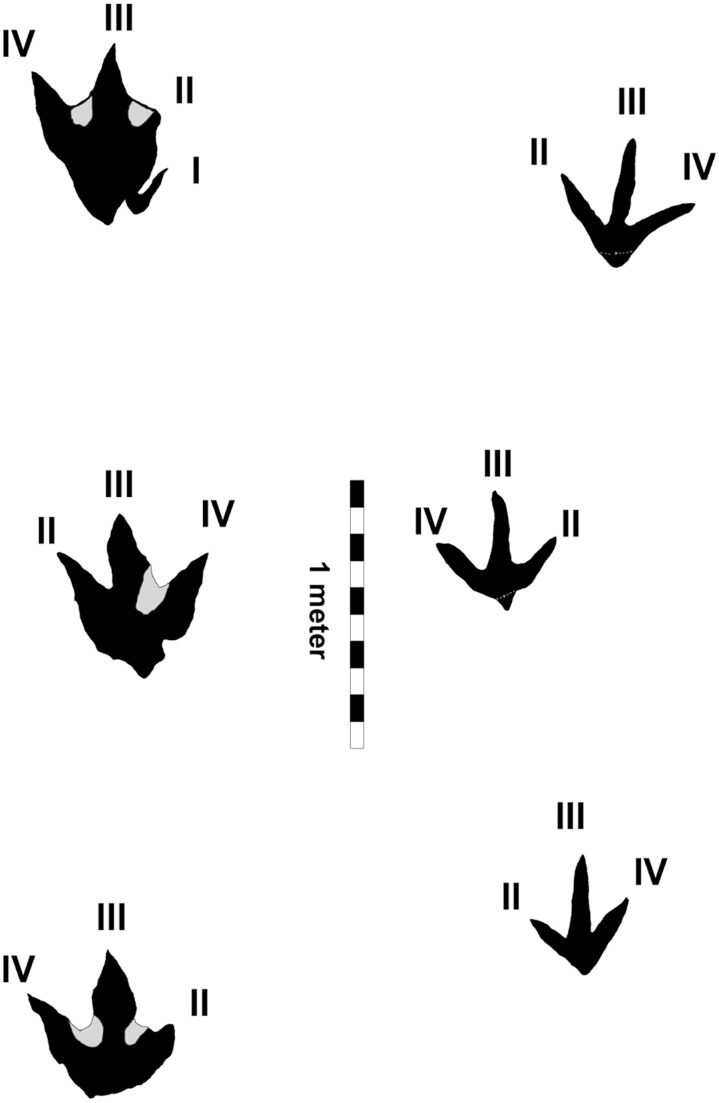
Comparison of (Footprint Area to Length Ratio). *Bellatoripes fredlundi* holotype, Trackway A, Print #2 PRPRC 2011.11.001 (left) Footprint Area to Length Ratio = 24.3 cm^2^∶1 cm. *Irenesauripus mclearni* track from the Gates Formation **(right)** Footprint Area to Length Ratio = 8.4 cm^2^∶1 cm.

**Figure 9:**
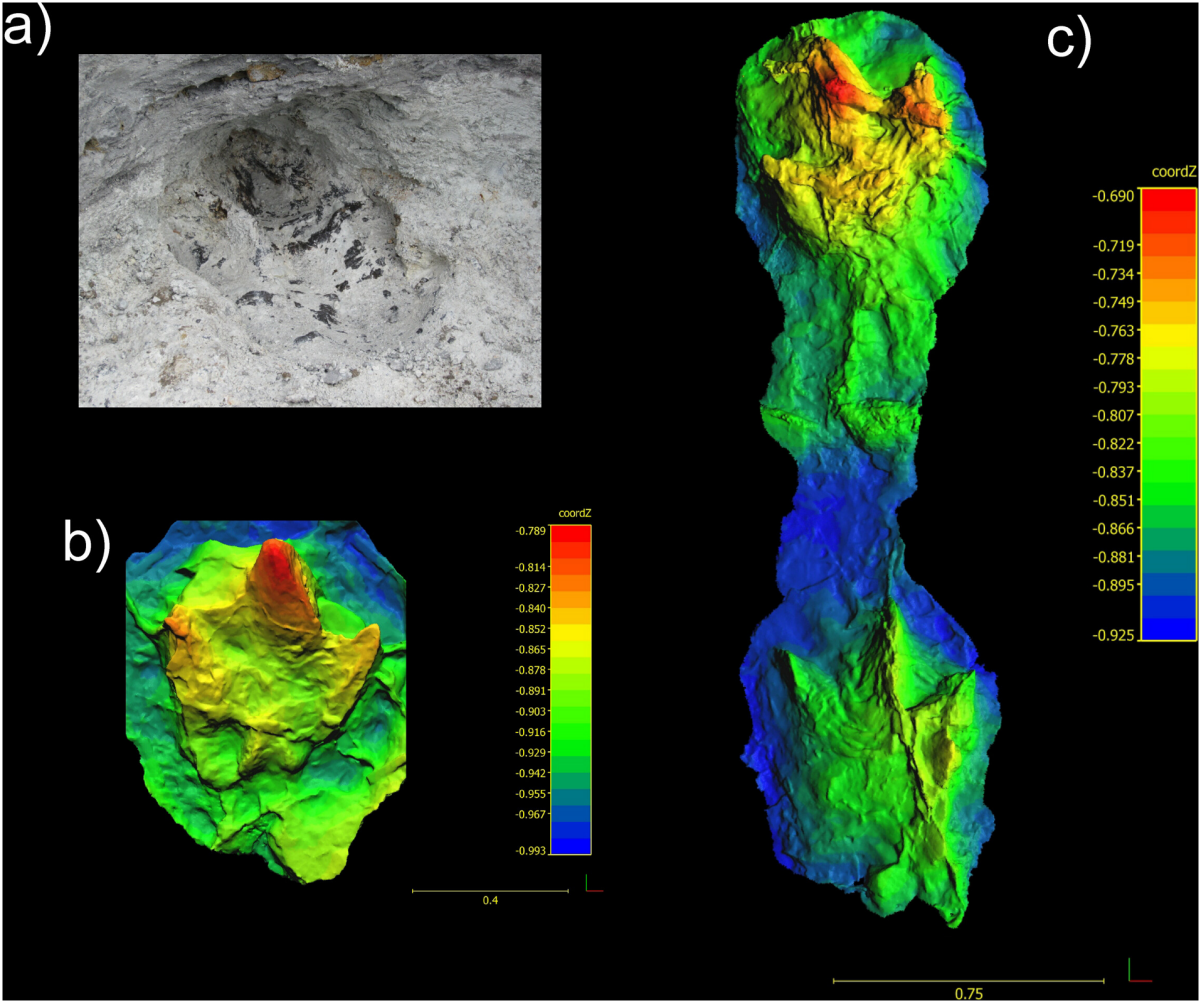
Trackway B of *Bellatoripes fredlundi*. a) Partially excavated print #2 of Trackway B (not mapped or moulded); b) Photogrammetric image of Trackway B, print #1 from its silicone mould (PRPRC 2012.04.002); c) Photogrammetric image of Trackway C, prints #1 and 2 from its silicone mould (PRPRC 2012.04.003). Topographic profile scales and linear scales are in meters.

*Bellatoripes fredlundi* ichnogen. et ichnosp. nov. (Figs. 1–3, 7–9)
(urn:lsid:zoobank.org:act:B64813F7-B142-4DB1-8DEF-0542E8D1E7DA).

### Etymology

*Bellatoripes*, bellatorius, Latin for “warlike”, “martial”, “pugnacious”; pes, Latin for “foot”; *fredlundi*, Latinized name in honor of Mr. Aaron Fredlund, who discovered and reported the type specimen in 2011.

### Diagnosis

Bipedal trackway of a large, functionally tridactyl, digitigrade track-maker. Footprints longer than wide with footprint length (FL) over 50 cm. Footprint Area to Length Ratio greater than 20 cm^2^∶1 cm. Prints are mesaxonic and digits II and IV are of similar length. Digit impressions are wide, lacking obvious digital pad impressions. Digits are thick proximally and taper strongly distally. The free digit length (FDL) of digit III is short compared to the length of the footprint. Total divarication is variable, but is generally no greater than 80^o^. Divarication between digits III and IV is normally greater than between digits II and III. Metatarsal pad impression present with a wide caudal margin. Pace length nearing 175 cm; stride length nearing 350 cm. Footprints in trackway are in line, with a pace angulation nearing 180^o^. Trackway width is narrow, with footprints overlapping the midline of the trackway. Footprint rotation is slight, but generally toward the trackway midline (Table 1).

**Table 1:** Footprint and trackway data for trackways A, B, and C of *Bellatoripes fredlundi*.

Trackway	Print	FL	FW	FR	DL			FDL			DW			DIV			D/MP D				DPA			PL	SL	PA
					II	III	IV	II	III	IV	II	III	IV	II–III	III–IV	TOT	II	III	IV	MP	II	III	IV			
A	1(L)	52.3	52.5	+6°	33.8	52.3	45.0	4.9*	29.2	24.1	10.7	16.4	12.5	38°	42°	80°	7.7	12.9	8.8	6.5	15°	20°	14°	-	-	-
	2(R)	62.0	51.5	–9°	54.8	62.0	52.0	23.5	29.3	18.5	14.3	15.5	14.5	24°	36°	60°	12.6	15.4	10.4	12.7	11°	20°	16°	174	-	-
	3(L)	59.5	51.8	+5°	40.8	59.5	58.4	2.8*	23.4	19.5	12.3	15.8	12.0	22°	29°	51°	13.2	12.6	5.4	9.6	20°	19°	7°	173	346	117°
	*X*	57.9	51.9	+2°	43.1	57.9	51.8	NA	27.3	20.7	12.4	15.9	13.0	28°	36°	64°	11.2	13.6	8.2	9.6	15°	20°	12°	173.5	346	117°
B	1(R)	67.0	52.5	-	59.5	67.0	50.3	17.2	32.6	22.4	10.5	17.5	22.4	30°	40°	70°	11.7	18.0	14.9	12.4	17°	27°	16°	-	-	-
	2(L)	-	-	-	-	-	-	-	-	-	-	-	-	-	-	-	-	-	-	-	-	-	-	-	-	-
C	1(R)	59.0	55.2	12°	49.3	59.0	52.4	24.8	34.2	30.8	15.2	16.2	12.0	32°	38°	70°	11.7	18.0	14.9	12.4	–24°	2°	–4°	-	-	-
	2(L)	62.0	56.0	20°	40.4	62.0	52.0	22.4	36.7	28.5	16.2	17.7	11.5	32°	29°	61°	13.7	17.4	18.0	13.5	12°	22°	7°	155	-	-

Linear measurements in cm. Data collected from PRPRC 2011.11.001M (Trackway A), PRPRC 2012.04.002M (Trackway B), PRPRC 2012.04.003M (Trackway C). DIV, digit divarication; FDL, free digit length; DD, digit depth; DL, digit length; DPA, digit plunge angle; DW, digit maximum width; FL, footprint length; FR, footprint rotation; FW, footprint width; L, left print; MP, metatarsal pad; MPD, metatarsal pad depth; R, right print; T, total divarication. PA, pace angulation; PL, pace length, PRPRC, Peace Region Palaeontology Research Centre; R, right; SL, stride length. *denotes digit lengths that are considered incomplete due to pathology. NA denotes mean data that is not available due to pathology.

### Holotype

*In situ* specimen PRPRC 2011.11.001 (Trackway A) natural mould with three pes prints (two left, one right); also replica silicone mold (PRPRC 2011.11.001M, Fig. 7) and fiberglass reinforced (FGR 95) plaster cast (PRPRC 2011.11.001MC) stored at the Peace Region Palaeontology Research Centre (PRPRC) collection, Tumbler Ridge, British Columbia. Print #2 (right) is designated as the holotype footprint.

### Paratypes

*In situ* specimens PRPRC 2012.04.002 (Trackway B, Figs. 9a–b); replica silicone mould (PRPRC 2012.04.002M) and PRPRC 2012.04.003 (Trackway C, Fig. 9c) and silicone mould (PRPRC 2012.04.003M).

### Referred Specimens

Print TMP 81.34.1 from the Belly River Group in Dinosaur Provincial Park, Alberta [6] and PRPRC 2004.08.001 from the Wapiti Formation of British Columbia [7].

### Type locality

Northeastern British Columbia, east of Tumbler Ridge. Precise locality on file at the Peace Region Palaeontology Research Centre, Tumbler Ridge, British Columbia, Canada.

### Type horizon

Upper Cretaceous (Campanian - Maastrichtian) Wapiti Formation, Unit 4 [26].

### Remarks

PRPRC 2011.11.001 is a 4 meter long trackway with three footprints (two left and one right) (Figs. 1–2, 7–8). The two left footprints each have an obviously truncated second digit, indicating pathology. The right footprint possesses three complete digit impressions (II–IV) and is designated as the holotype. Entry striations and skin tubercle impressions have been found in each footprint. Striations and some skin tubercle impressions are found along one or both sides of digit III on all prints, and skin tubercle impressions are found in the metatarsal pad area of print #3. The presence of skin impressions indicates that these are true tracks, rather than undertracks.

A list of synapomorphies that are potentially observable in footprints was proposed by Carrano and Wilson [27]. Features present in the *Bellatoripes fredlundi* tracks and trackways, such as bipedal trackway, narrow gait, lack of metatarsal prints, and a laterally divergent digit IV indicate that *Bellatoripes fredlundi* shares some features in common with Dinosauromorpha, Dinosauriformes and Dinosauria. The presence of clawed digit impressions with prints reflecting mesaxonic, functionally tridactyl pedes indicates the track-makers belong to Theropoda. There are no recognized feather traces associated with the *Bellatoripes fredlundi* tracks, which is presently the only synapomorphy that could support inclusion of this ichnotaxon within Coelurosauria [27]. Tyrannosauroidea and Tyrannosauridae synapomorphies [28] do not include features of the feet that could be reflected in tracks. Farlow *et al.* [29] comment that in order for many of the synapomorphies listed by Carrano and Wilson [27] to be impressed in a substrate the track-maker would have to sit on the ground. The argument for the identification of *Bellatoripes fredlundi* tracks being made by tyrannosaurids is of necessity based on footprint size, overall morphology (although lacking presently known synapomorphies of Tyrannosauroidea and Tyrannosauridae) as well as stratigraphic and geographic occurrence. These observations reasonably exclude any other theropod clade as potential track-makers for *Bellatoripes fredlundi*. Furthermore, *Bellatoripes fredlundi* tracks, in common with other footprints, are impressions of feet that possessed skin, muscle, tendons and other tissues supported by a skeletal framework. Soft tissue anatomy is only very rarely preserved with body fossils, thus synapomorphies of Tyrannosauroidea and Tyrannosauridae are, out of necessity, based on skeletal features. The characteristic of digits impressions thickened proximally and strongly tapering distally in Tyrannosauripodidae is a reflection of the soft tissue anatomy of the foot of the track-maker and is unique to this ichnofamily and the included ichnotaxa.

If the identification of ichnotaxa within Tyrannosauripodidae as tyrannosaurid ichnotaxa stands the test of time and scientific consensus, some of the characteristics of this ichnofamily may become synapomorphies of Tyrannosauroidea and Tyrannosauridae.

Farlow *et al.* [29] indicate the difference in the greater free length of digit IV compared with digit II as having potential for a tyrannosaurid-specific feature that could be preserved in tracks. This feature was observed by Farlow *et al.* [29] in tracks from Alberta described by McCrea *et al.* [6]. Specimens of *Bellatoripes fredlundi* show equivocal results for this proposed character. Out of a population of six measured footprints (Table 1) only four prints were useful due to a repetitive pathology of digit II of prints #1 and #3 for Trackway A. The intact print #2 of Trackway A displays a digit II with longer free digit length (FDL) than digit IV. Digit IV has a free digit length greater than that of digit II in the single measured print of Trackway B. Digit II free digit length measurements of both prints of Trackway A were greater than the digit IV measurements. This osteological character may simply be masked by the soft tissues of the track-makers’ feet.

## Comparative Ichnology

Morphological characteristics of *Bellatoripes fredlundi* tracks and characteristics of their trackways are markedly distinct from other previously described large theropod ichnotaxa. *Tyrannosauripus pillmorei* [4], almost certainly a track of *Tyrannosaurus rex*, is the only ichnotaxon that is comparable to *Bellatoripes fredlundi*, as it is similarly robust with digit impressions that are wide proximally and taper strongly distally. *Tyrannosauripus pillmorei* prints are substantially longer (40%) and wider (20%) than those of *Bellatoripes fredlundi*. *Tyrannosauripus pillmorei* also has indistinct digital pad impressions and a hallux that is directed medially, whereas *Bellatoripes fredlundi* has no digital pad impressions and a cranially-directed hallux impression. Trackway characteristics of *Tyrannosauripus pillmorei* are unknown as the only specimen is a single natural cast footprint from the Raton Formation (Upper Cretaceous-Paleocene) several meters below the Cretaceous/Paleogene boundary.

A few tracks from Alberta have been identified as those of tyrannosaurids [6], [8], [29]. One of these (TMP 81.34.1, fig. 21.4 in McCrea *et al.* [6]) was initially identified as an ornithopod print, but later identified as a tyrannosaurid print [16], [29]. TMP 81.34.1 is quite similar to *Bellatoripes fredlundi*, with a 56.2 cm length and proximally wide digits (digit widths: II-16.2 cm, III-17.2 cm, IV-16.0 cm, table 21.1 in McCrea *et al.* [6]) which taper to points. This specimen has been referred to *Bellatoripes fredlundi*. Other prints (TMP 93.36.282) described by McCrea *et al.* [6] and UALVP 53475 reported by Fanti *et al.* [8]) are large and were probably produced by tyrannosaurids. These specimens (TMP 93.36.282 – figs. 21.5–21.7 [6], and UALVP 53475 fig. 5 [8]) lack the robustness of *Bellatoripes fredlundi*, as well as possessing noticeable digital pad impressions which are absent in both *Bellatoripes fredlundi* and *Tyrannosauripus pillmorei*. These prints are smaller than *Bellatoripes fredlundi* (TMP 93.36.282 is 51.9 cm; and UALVP 53475 is 49.0 cm) with digit widths nearly half that reported for *Bellatoripes fredlundi*, and may represent an earlier ontogenetic stage of the tyrannosaurids that were likely the makers of tracks of *Bellatoripes fredlundi*.

Calculations estimating femur length from the length of the type footprints (see Materials and Methods, Figs. 5–6) suggest the approximate ages of the tyrannosaurid track-makers of *Bellatoripes fredlundi* Trackways A, B, and C were 26, 29, and 25 years (+/–2 years), respectively. The estimated age for the maker of Trackway B is near the upper age-limit estimated for tyrannosaurids [21], [30], [31], indicating the track-maker was fully adult, although such age-limits may be minimum estimates [32]. The track-maker of TMP 93.36.282 is calculated have been approximately 21 years of age and the track-maker of the smaller UALVP 53475 was approximately 20 years of age. These smaller tyrannosaurid tracks (UALVP 53475 and TMP 93.36.282) have a greater overall similarity to many track types that have been identified as allosaurid from older sedimentary deposits. This observation fits with observations [30], [31] that juvenile tyrannosaurs were much more gracile than adult tyrannosaurs. Juvenile tyrannosaurids may have retained a gracile body form to a certain age, after which they fleshed-out to a robust adult form. There are much more specimens and data that need to be collected before an attempt is made to construct an ontogenetic series for tyrannosaurids based on footprints, but it is possible that the timing of this transition from gracile juvenile to robust adult may be found by studying tyrannosaur footprints. In the meantime, it would be prudent to be cautious about naming new ichnotaxa based on track material that may have been produced by juvenile tyrannosaurids.

Tracks of *Irenesauripus mclearni* [33] are much less robust than those of *Bellatoripes fredlundi*. The surface area of print #2 of Trackway A of *Bellatoripes fredlundi* was calculated as 1509 cm^2^. When divided by footprint length (62.0 cm), the Footprint Area to Length Ratio was calculated as 24.3 cm^2^∶1 cm. For comparison, a large theropod print (*Irenesauripus mclearni*, a presumed allosauroid) from the Gates Formation (Lower Cretaceous: Albian) was found to have a surface area of 483 cm^2^. When divided by its footprint length (57.5 cm) the Footprint Area to Length Ratio of the *Irenesauripus mclearni* print is 8.4 cm^2^∶1 cm, which is substantially less than the ratio calculated for print #2 of *Bellatoripes fredlundi*. The Footprint Area to Length Ratio quantitatively demonstrates that the track of *Bellatoripes fredlundi* is more robust than the print of *Irenesauripus mclearni* (Fig. 8). Tracks of the *Irenesauripus mclearni* holotype (CMN 8548) possess no hallux impressions, and no hallux impressions have been observed to date in any other tracks that have been attributed to *Irenesauripus mclearni* from western North America. Digital pad impressions for this ichnotaxon are usually well-developed on the comparatively long and slender digit impressions. The *Irenesauripus mclearni* holotype specimen is significantly smaller (FL: 38 cm) than those of *Bellatoripes fredlundi*, although some prints identified as *Irenesauripus mclearni* from western Canada are up to 50 cm length. *Irenesauripus mclearni* trackways have much shorter pace (94 cm for the holotype trackway) and stride values with lower pace angulation. Furthermore, they frequently show greater footprint rotation towards the midline than the *Bellatoripes fredlundi* holotype, but usually less footprint rotation than observed in the *Bellatoripes fredlundi* paratype (Trackway C).

The holotype of *Irenesauripus acutus* (CMN 8549) is much larger (FL: 53.5 cm) than the *Irenesauripus mclearni* holotype [33] and is similar in size to some tracks of *Bellatoripes fredlundi*. However, *Irenesauripus acutus* tracks have not been observed to have hallux impressions. The footprints of *Irenesauripus acutus* are proportionally longer and more slender than those of *Irenesauripus mclearni*, and though not evident in Sternberg’s [33] figure 2 illustration [33], *Irenesauripus acutus* tracks possess well-developed digital pad impressions as evidenced by a photograph of an *Irenesauripus acutus* track on pg. 81, Plate 3 of Sternberg [33]. The measured pace of the *Irenesauripus acutus* holotype is 173 cm, which is within the range found with *Bellatoripes fredlundi* trackways.

Prints of *Bueckeburgichnus maximus* described from the Lower Cretaceous of Germany [34], [35] are similar in size (FL: 56 cm) to *Bellatoripes fredlundi*. *Bueckeburgichnus maximus* digit impressions are not as robust as those of *Bellatoripes fredlundi* and the digit impressions possess traces of digital pads, although Lockley [35] noted that digit II of *Bueckeburgichnus maximus* was similar to that of *Tyrannosauripus pillmorei* in being broad and well-padded. *Bueckeburgichnus maximus* tracks have a proportionally short, medially-directed hallux impression. A *Bueckeburgichnus maximus* track also possesses a narrow caudal margin of the metatarsal pad area (the heel) whereas *Bellatoripes fredlundi* tracks have a much wider metatarsal pad trace, sometimes with two or three lobe-like impressions (Fig. 9b). As *Bueckeburgichnus maximus* was described from a single print [34], [35], its trackway characteristics are unknown.

A description of *Megalosauripus* (*Megalosauripus uzbekistanicus*, *Megalosauripus teutonicus* and other specimens assigned to this ichnogenus) by Lockley *et al.* [36] shows that these are large theropod tracks (FL: 40–77 cm) with a wide global and stratigraphic (Upper Jurassic to Lower Cretaceous) distribution. A *Megalosauripus* track differs considerably from a *Bellatoripes fredlundi* track in having observable digital pad impressions (although not observed in *Megalosauripus teutonicus*), and a narrow, elongate heel impression. Where present, hallux impressions are proportionally short and directed caudomedially to craniomedially. *Megalosauripus* trackway characteristics are distinctly irregular and variable, and generally display low pace angulation, although some *Megalosauripus* trackways from North America are known to have high pace angulation values (175^o^) [36].

*Eutynichnium lusitanicum* is a large theropod ichnotaxon (FL: 37–40 cm excluding hallux) described from the Late Jurassic of Portugal [36]. Digit impressions II–IV are wide, but do not taper, differing from those of *Bellatoripes fredlundi* which are wide and taper strongly to acute points. Digital pad impressions are not clearly defined, which is somewhat similar to *Bellatoripes fredundi* specimens where they are absent. A *Eutynichnium lusitanicum* print had a hallux impression that is directed craniomedially, similar to that of *Bellatoripes fredlundi* when present. The caudal margin of the metatarsal pad impression of *Eutynichnium lusitanicum* is narrow compared to that of *Bellatoripes fredlundi*. *Eutynichnium lusitanicum* has a relatively short pace (100 cm) compared with *Bellatoripes fredlundi* where the pace varies between 155 cm (Trackway C) and 174 cm (Trackway A). The difference in size between the track-makers of *Eutynichnium lusitanicum* and *Bellatoripes fredlundi* may account for some of the difference in pace length; however, the pace lengths observed in *Eutynichnium lusitanicum* are shorter than those observed in similar sized theropod footprints such as *Irenesauripus mclearni* [33].

*Eubrontes* [37] is an ichnogenus of tracks from the Lower Jurassic, attributable to large theropods, with maximum footprint lengths just over 40 cm. *Eubrontes* tracks, unlike *Bellatoripes fredlundi*, often possess obvious digital pad impressions [38], [39], as well as narrow caudal margins of the metatarsal pad area. Recently described specimens identified as *Eubrontes* from the Lower Jurassic of Utah show a craniomedially-directed hallux impression [40], although hallux traces are not evident in many previously described and figured specimens. *Eubrontes* trackways tend to have a maximum pace length of just over 1.2 meters with footprint rotations varying between 0^o^ and 10^o^ towards the midline of the trackway [39].

*Gigandipus* [41], [42] is a large theropod ichnogenus described from the Lower Jurassic that differs from *Bellatoripes fredlundi* in possessing well-defined digital pad impressions, a more medially-directed hallux impression and a narrow caudal margin of the metatarsal pad trace. The footprint length of *Gigandipus* is just under 45 cm [38], [39]. Lull [38] gave a pace value of just over one meter for *Gigandipus* and footprint rotations varying between 0^o^–10^o^ towards the midline of the trackway [39]. From the diagram of a *Gigandipus* trackway (fig. 52 [39]) the pace angulation appears to be close to 180^o^, similar to that described for Trackway A of *Bellatoripes fredlundi*.

## Inferred Track-maker

The only specimens of theropods recovered from Upper Cretaceous (upper Campanian - lower Maastrichtian) deposits of western Canada with pedes large and robust enough to produce tracks of *Bellatoripes fredlundi* are those of tyrannosaurids, (the albertosaurines *Albertosaurus* or *Gorgosaurus*, and the tyrannosaurine *Daspletosaurus* [28]). Given the present osteological record, it is unlikely that these traces were made by an as yet unreported large Upper Cretaceous non-tyrannosaurid theropod, such as giant ornithomimosaurians or oviraptorosaurians. Giant ornithomimosaurians are reported from the Early Cretaceous of China but were likely functionally tetradactyl [43], and those reported from North America [44] are too small to have made these tracks**.** Many specimens of oviraptorosaurians possess a comparatively long digit I [45] and were likely functionally tetradactyl [46], [47]. Large-bodied oviraptorosaurians described to date from North America [48] are also too small (∼3.50 m body length) to be a potential track-maker of *Bellatoripes fredlundi*.

### Unusual Features

There is an inferred pathology associated with digit II of the left footprint (seen in prints #1 and #3 of PRPRC 2011.11.001, Trackway A), reducing the length of digit II by at least 14 cm (Table 1; Figs. 4, 7–8). The pathology may have involved the loss of the distal and penultimate phalanges (II-2, II-3), or it may be a trace of a deformation or dislocation that prevented the distal portion of digit II from contacting the substrate. The rough, uneven margin of the distal ‘nub’ of the digit II impression is consistent with a wound that would have involved a loss of tissue and bone.

The pathology on digit II (left prints) of Trackway A does not appear to have significantly impaired the track-maker’s locomotion. Stride, pace and pace angulation values indicate a normal and efficient gait for a large theropod. The pes rotation of the second print (right) is the opposite of what is normally expected (outward rather than inward rotation from the midline of the trackway), and is consistent with compensation for this injury or deformity. This specimen is an interesting addition to the growing literature of dinosaur footprint pathology [49] and to the literature of tyrannosaurid-specific pathologies [50], [49].

### Locomotion and behavior

**Figure 10:**
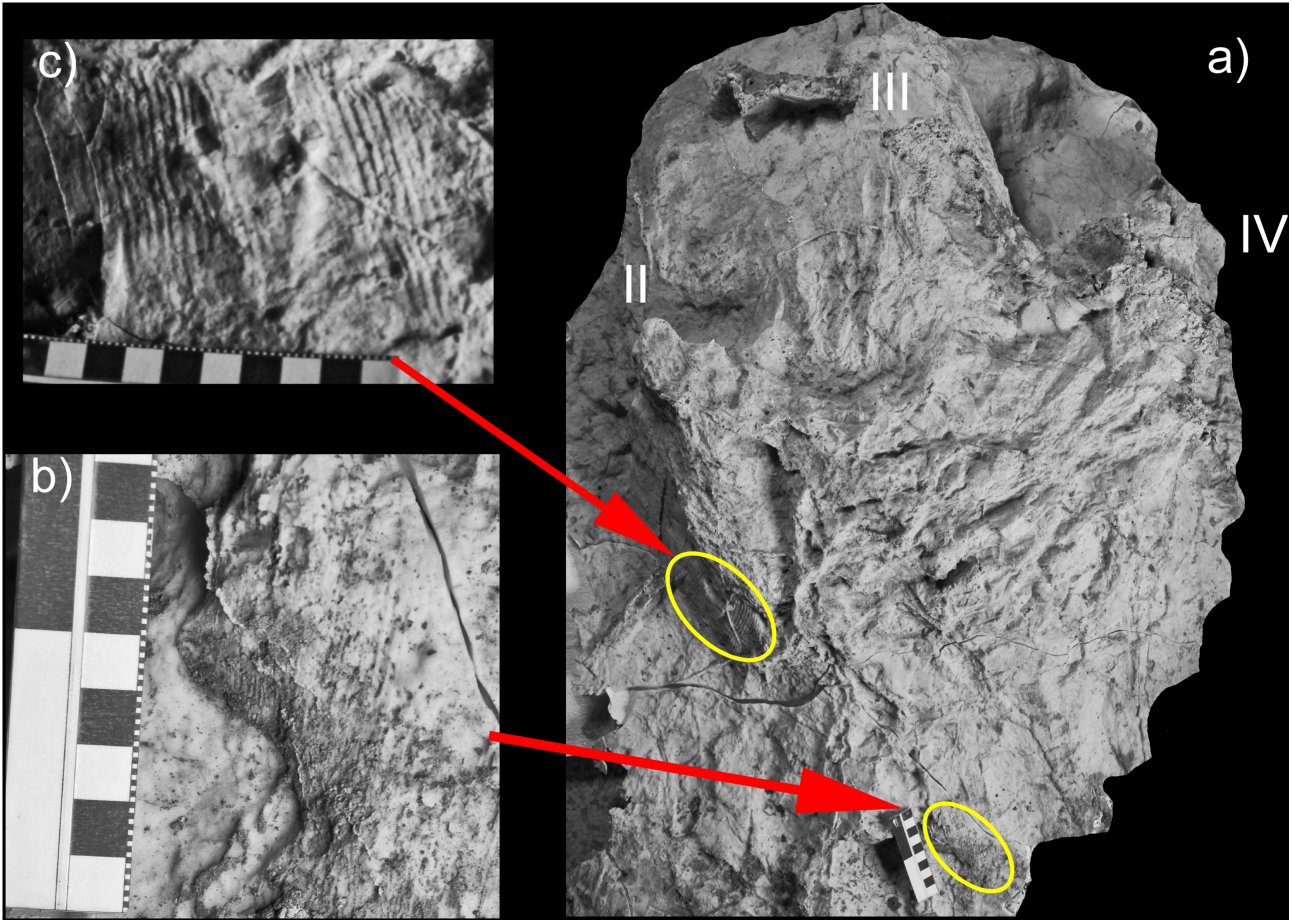
Striations on *Bellatoripes fredlundi* paratype track. a) Photograph of silicone mold of print #2 Trackway C (PRPRC 2012.04.003), arrows pointing to areas with striations; b) photograph of striations on caudal drag marks leading up to print #2; c) photograph of striations on the outer margin of digit II print #2 Trackway C.

The individual footprints in PRPRC 2011.11.001 (Trackway A), the first print of PRPRC 2012.04.002 (Trackway B) and the second print of PRPRC 2012.04.003 (Trackway C) undercut the original surface of the track-bearing layer to a considerable degree. The skin impressions provide information on foot movement [51] and in addition are evidence of true tracks that are not compromised by poor preservation. Two areas of lengthy (7.2 cm and 12.0 cm lengths), parallel striations (4 striations per cm width perpendicular to the long-axis of the striations) are present on shallow drag marks leading up to the caudal edge of print #2, Trackway C (Fig. 10). Both sets of impressions are parallel to the direction of travel (7^o^ toward digit II in relation to the central axis of digit III with a 10^o^ plunge in the direction of travel). They are interpreted as being the result of the caudal portion of the foot making shallow contact with the substrate (pre-touch-down phase) as the foot moved forward towards the area where the foot begins to settle (touch-down phase) prior to the animal putting its full weight down (weight-bearing phase). The footprint cycle ends with the kick-off phase [52] leading towards the next touch-down phase. These striations are therefore identified as entry striations.

A third area of striations (4 striations/cm width) is present on the same footprint, but occurs along a 27 cm long portion of the outer edge of digit II. In relation to the rest of the footprint these striations extend from the surface of the substrate and run in an anterior (7.3 cm) and downward (6.2 cm) direction, representing a 50^o^ plunge angle, 35^o^ towards digit IV in relation to the central axis of digit III. The striations are deeply incised into the substrate on the caudal portion of the digit II impression, but become less distinct toward the cranial portion of this digit. These are interpreted as entry-striations recording the trajectory of the foot entering the substrate and quickly penetrating to the deepest point of the weight-bearing phase [52]. As the foot settled it slipped forward and away from the midline of the trackway. Alternatively, the digit II striation impressions could be interpreted as exit striations recording the trajectory of the foot as it is withdrawn backwards and slightly towards the trackway midline. The process of limb movement we propose below likely fits with this latter interpretation.

**Figure 11:**
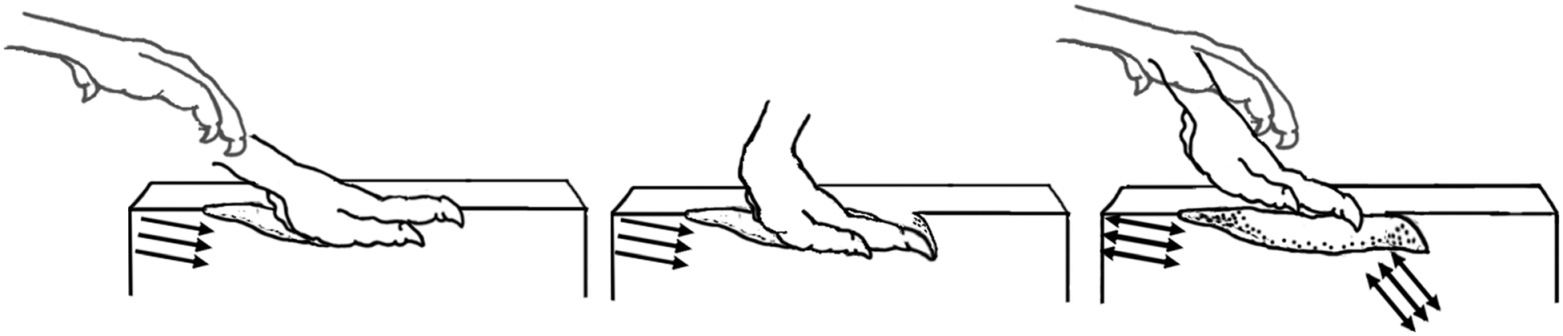
Hypothesized pes movement of the track-maker for *Bellatoripes fredlundi*. The digits were not dragged cranially through the substrate as previously described in theropods footprints [53], [54], [55]. The pes and digits were retracted from the substrate along an opposite trajectory of their entry prior to the pes moving forward in the next step cycle. Arrows indicate trajectory of the foot of Trackway C, print #2 as deduced from entry striations at the point of pes entry (left frame) and exit (center frame) into the substrate.

The foot movements deduced by study of the *Bellatoripes fredlundi* tracks contrasts with published observations of trackways of Triassic [53], Jurassic [54], and Cretaceous [55] theropods in which the footprints indicate that the track-makers’ feet were dragged cranially out of the substrate after registering during the kick-off phase [52]. This could be explained as a function of the depth of the substrate compared to the size of the animals involved. The difference may also be related to the consistency of the substrate: the substrate consistency for the reported Triassic prints [53] may have influenced the movement of the limbs such that caudal withdrawal of the pedes from the substrate was not possible, in contrast to the reported prints of Jurassic and Late Cretaceous theropod tracks [56], [57]. In the extant *Struthio camelus*, flexion and extension of the intertarsal and metatarsophalangeal joints are assisted by the elastic energy storage/release by ligaments, and tendons of the gastrocnemius and digital flexor muscles [58]: during a full-motion cycle, flexion of the intertarsal joint occurs automatically with tarsometatarsal abduction, while extension results in adduction [58] (the passive engage-disengage mechanism of Schaller *et al.* [59]). The track-makers of *Bellatoripes fredlundi*, while utilizing hip-driven rather than knee-driven locomotion [60], [61], may have consequently withdrawn their pedes during intertarsal flexion enough to retract the digits and remove them from the footprint caudally, instead of dragging the digits cranially through the substrate (Fig. 11). The claw impressions of several of the *Bellatoripes fredlundi* tracks from the British Columbia site undercut the substrate and were not disturbed by the withdrawal of the feet. This is consistent with the interpretation of the withdrawal of the foot caudally from the footprint during the take-off phase of the footprint cycle.

In recent years, the evidence for gregarious behaviour in some species of theropod dinosaurs has substantially increased [14], [15], [62], [63]. This evidence takes the form of osteological signaling features and pathologies that show interactions between individuals, and analysis of monodominant bonebeds [15]. Although tyrannosaurid footprints are rare, it is significant that the first reported tyrannosaurid trackways represent three individuals moving in the same direction (roughly southeast: Trackway A –116°, Trackway B –120°, and Trackway C –128°, unadjusted) in close proximity (just over 5.5 meters between Trackways A and B, and just over 2.5 meters between Trackways B and C). Given that tyrannosaurids normally make up only five percent of the faunal composition [64], the probability of three unassociated tyrannosaurids walking in parallel (8.4 m between the two individuals that are farthest apart) is low. The preservation (depth of impression, lack of compression uplifts, evidence of skin impressions and striations) of the footprints in all three trackways suggests that they were made at approximately the same time, and increases the likelihood that these track-makers were associated. Tracks and trackways of smaller theropods and large ornithopods at this tracksite do not follow the same bearing as the tyrannosaurid trackways. In fact, the non-tyrannosaurid trackways are random in regards to compass bearing, which rules out a geographic barrier that might have compelled the tyrannosaurids to walk in the same direction and in close association. The inference that these three animals were moving as a social group is the most parsimonious interpretation based on current data [15] and provides the first trackway evidence showing gregarious behaviour in tyrannosaurs.

In calculating the relative velocity for the track-maker of *Bellatoripes fredlundi*, for Trackway A the estimated *h* of the track-maker is 2.87 m (+/–1.30 cm). It is worth pointing out that *h* calculated using the general large theropod equation [17] is 2.80 m. While the *h* for tyrannosaurids may not be significantly different from results obtained from the original equation [17], [18], the opportunity exists to determine the hip height to footprint length relationship for other taxa of both small and large theropods, which may be of use in studies that combine both computer simulations and known trackways.

One issue with using *h* to calculate relative velocity as per Thulborn and Wade [17] is that it only takes into account a completely straight hind limb, which is not anatomically accurate. The straight-leg hip height provides a lower limit to the velocity of the track-maker of *Bellatoripes fredlundi*. Taking into account a flexion of the knee of 110° and a flexion at the ankle of 140° [65], [66], an estimated hip height of 2.30 m is obtained. Using both values for *h* results in a range of relative velocities from 6.40 km/hr to 8.50 km/hr (+/–0.40 km/hr) for the maker of Trackway A, with a stride length (λ) to *h* ratio of 1.69 at the lower end of the range. This correlates with the hypothesized energetically optimal walking gait as determined for large bipedal dinosaurs [23], although the concept of such an optimum has been disputed [61]. Trackway A likely represents the optimal gait that the tyrannosaurid track-makers habitually used for general locomotion. While there is the need for caution in using footprint length in calculating track-maker velocities [67], the *Bellatoripes fredlundi* trackways comprise true tracks which are the least susceptible to erroneous velocity estimates.

The locomotory capabilities of tyrannosaurids have been studied and discussed at length in numerous articles [10], [60], [61], [68], [69], [70], [71], [72], [73], [74], [75], [76], [77], mostly addressing their speed and agility (specifically that of *Tyrannosaurus rex*). The calculated relative velocity of the tyrannosaurid track-maker of Trackway A of *Bellatoripes fredlundi* shows an animal that was moving at a typical walking gait, and does not provide insight into the top speed of the carnivorous track-maker. However, the trackways of *Bellatoripes fredlundi* provide the first record of the walking gait of tyrannosaurids. If tyrannosaurids were capable of higher-velocity gaits these would likely be at higher velocities than 8.50 km/h. Future models testing the potential velocity and gait of a tyrannosaurid will have the opportunity to incorporate the preserved track record of this group as an analytical control.
