# Correction: A ‘Terror of Tyrannosaurs’: The First Trackways of Tyrannosaurids and Evidence of Gregariousness and Pathology in Tyrannosauridae

**DOI:** 10.1371/journal.pone.0117606

**Published:** 2015-01-09

**Authors:** The PLOS ONE Staff

Reference 65 is a duplicate of reference 11. All citations of reference 65 in the text of the article should instead cite reference 11. All other citations and references are correct.
